# Topological transitions in an oscillatory driven liquid crystal cell

**DOI:** 10.1038/s41598-020-75165-8

**Published:** 2020-11-09

**Authors:** Marcel G. Clerc, Michał Kowalczyk, Valeska Zambra

**Affiliations:** 1 Departamento de Física and Millennium Institute for Research in Optics, FCFM, Universidad de Chile, Casilla 487-3, Santiago, Chile; 2 Departamento de Ingeniería Matemática and Centro de Modelamiento Matemático (UMI 2807 CNRS), Universidad de Chile, Casilla 170 Correo 3, Santiago, Chile

**Keywords:** Nonlinear phenomena, Phase transitions and critical phenomena, Nonlinear optics

## Abstract

Matter under different equilibrium conditions of pressure and temperature exhibits different states such as solid, liquid, gas, and plasma. Exotic states of matter, such as Bose–Einstein condensates, superfluidity, chiral magnets, superconductivity, and liquid crystalline blue phases are observed in thermodynamic equilibrium. Rather than being a result of an aggregation of matter, their emergence is due to a change of a topological state of the system. These topological states can persist out of thermodynamics equilibrium. Here we investigate topological states of matter in a system with injection and dissipation of energy by means of oscillatory forcing. In an experiment involving a liquid crystal cell under the influence of a low-frequency oscillatory electric field, we observe a transition from a non-vortex state to a state in which vortices persist, topological transition. Depending on the period and the type of the forcing, the vortices self-organise, forming square lattices, glassy states, and disordered vortex structures. The bifurcation diagram is characterised experimentally. A continuous topological transition is observed for the sawtooth and square forcings. The scenario changes dramatically for sinusoidal forcing where the topological transition is discontinuous, which is accompanied by serial transitions between square and glassy vortex lattices. Based on a stochastic amplitude equation, we recognise the origin of the transition as the balance between stochastic creation and deterministic annihilation of vortices. Numerical simulations show topological transitions and the emergence of square vortex lattice. Our results show that the matter maintained out of equilibrium by means of the temporal modulation of parameters can exhibit exotic states.

## Introduction

Solid, liquid, gas, and plasma are different states of the matter^1^ distinguished from each other by mechanical, optical, and other properties. Other examples of states of aggregation of matter include glassy and liquid crystal states. Still different are exotic states such as Bose–Einstein condensates^2^, superfluidity^3^, superconductivity^6^, chiral magnets^4^, and liquid crystalline blue phases^5^ that are a topological state rather than an aggregation of matter. The topological transitions of the matter were discovered at the beginning of the 70s by Berezinskii^7^ and Kosterlitz and Thouless^8^, who showed that a low dimensional system described by a physical vector order parameter in thermodynamic equilibrium undergoes a transition from a homogeneous state without vorticity to a state in which vorticity persists. In the homogeneous state all vectors are unidirectionally ordered but under suitable conditions they realign forming regions where both their orientations and magnitudes vary. Because of topological constraints at some isolated points called vortices^9^ the vector field vanishes and the vector phase becomes undefined. The winding number (topological charge) is introduced to characterise the physical vector field around a vortex^9^. This number is an integer representing the total number of times that the vector field winds around the origin while varying along a closed, counterclockwise oriented curve around the singular point. Topological stability of the system implies that the total winding number of the system must be preserved which means that the vortices are created or annihilated by pairs of opposite topological charges. Vortices creations and and annihilation process are, respectively, due to thermal fluctuations and free energy minimisation^10,11^, hence at a critical temperature at which they are balanced the systems undergoes a topological transition^7,8^. Exotic states of matter such as Bose–Einstein, superconductivity, chiral magnets, and superfluidity are usually observed at low temperatures, however liquid crystalline blue phases have been observed at room temperature^12^.

**Figure 1:**
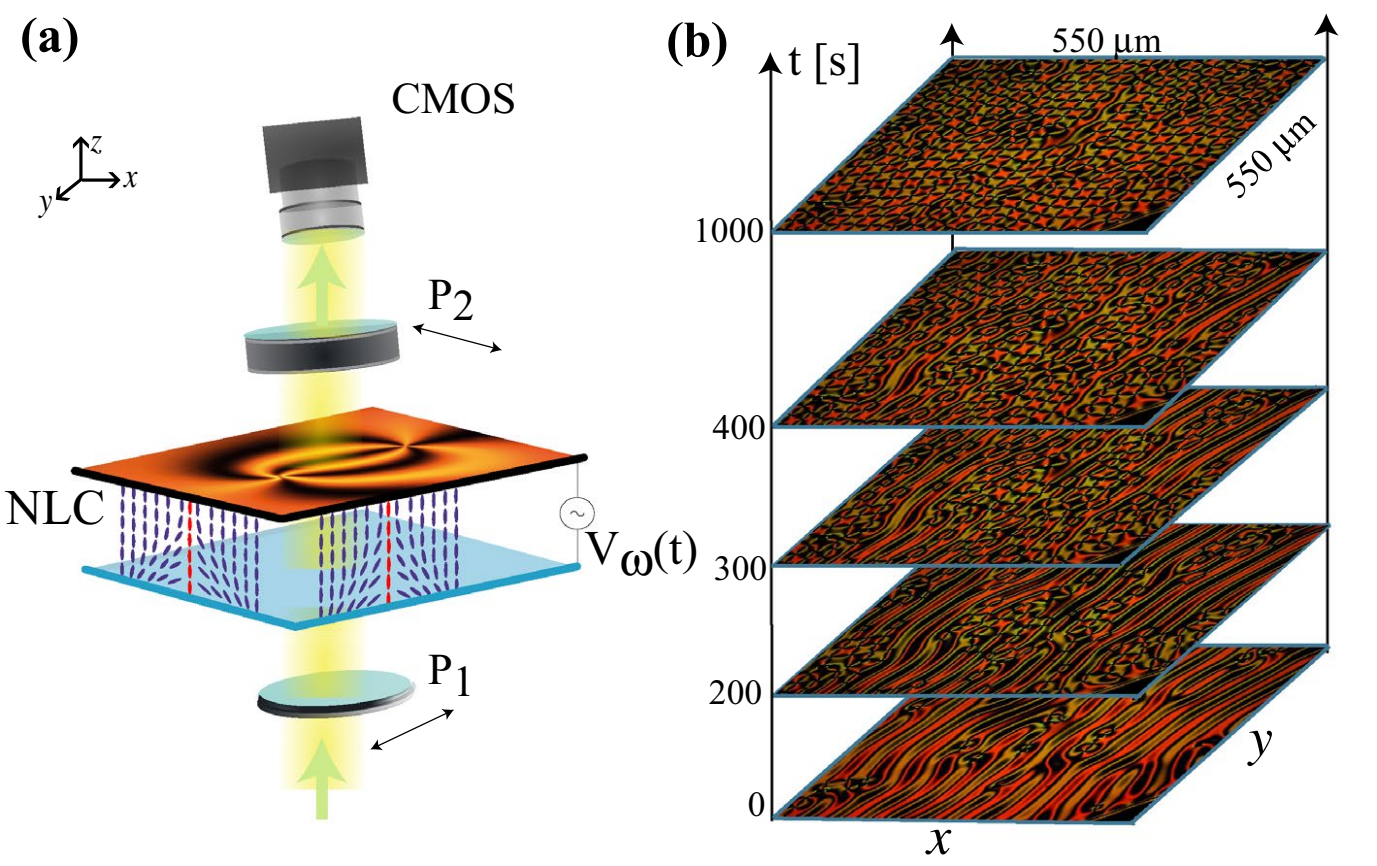
Liquid crystal cell under a temporarily modulated potential exhibits creation and self-organisation of vortices. (**a**) Schematic representation of the experimental setup. Liquid crystal cell (NLC) with homeotropic anchoring is illuminated by white light between two crossed polarisers ($P_1$ and $P_2$). The horizontal snapshot shows a pair of vortices with opposite charges. The purple and red rods illustrate the average molecular orientation (director) and the core of the umbilical defect. (**b**) The temporal sequence of snapshots in the region of self-organised vortices, at frequency 0.335 Hz and voltage amplitude 13.5 Vpp. From experimental snapshots, both figures were created using Inkscape 1.0.

An ideal material to investigate vortex dynamics are liquid crystals in thin films^5,13^. Liquid crystal cells under the effect of electric, magnetic, and electromagnetic fields can exhibit rich self-organisation such as patterns, traveling waves, defect dynamics, and spatiotemporal chaos (see review ^14^ and references therein). One of the most studied vortices are the so-called umbilical defects or disclination lines^5,13,15^. In thermodynamic equilibrium and homogeneous media, the vortices tend to annihilate by pairs to minimise the free energy of the system. The above dynamics can be modified by means of incorporation of inhomogeneities, which can attract and trap umbilical defects^16,17^. Properly distributed inhomogeneities may permit the formation of topology lattice^16^. Likewise, considering inhomogeneous anchoring allows attracting and trapping umbilical defects and creating vortex lattices^18,19^. A similar effect can be achieved by the introduction of inhomogeneous electrodes^20–23^. The combined use of magnets and uniform electric field can induce umbilical defects and lattices^24^. The vortex lattices describe above are induced by the combination of the forcing and inhomogeneities. However, the emergence of spontaneous topological lattices has also been achieved by means of thermal gradients^25^ or by doping with ionic impurity^21^, which induces charge motions. This is known as the Carr-Helfrichh mechanism^5^. The movements of charges of the liquid crystal or incorporated (ions) can be responsible for the formation of spatial structures (see Rev. ^14^ and references therein).

This article aims to study topological transition with injection and dissipation of energy by means of oscillatory forcing. This type of physical context usually is denominated as out of equilibrium systems^26,27^. Based on an experiment involving a nematic liquid crystal cell under the influence of a low-frequency oscillatory electric field, we observe a transition from non-vortex state to a state in which vortices persist, topological transition. Depending on the frequency and the type of the forcing, the vortices self-organise forming square lattices, glassy states, and disordered vortex structures. Sawtooth and square forcing induce a continuous topological transition characterised by the emergence of vortices with a disordered structure. Theoretically, a stochastic amplitude equation allows us to reveal the origin of the transition in terms of the balance between stochastic creation and deterministic annihilation of vortices. The above scenario changes drastically, when considering harmonic forcing, the observed topological transitions are characterised by the emergence of regular square vortex lattices (discontinuous transitions). By modifying the voltage frequency, we observe discontinuous transitions to different regular lattice and to other types of glassy vortex lattices.To account for these intriguing phenomena, we have included inertia in the amplitude equation. Numerical simulations show the emergence of square vortex lattices.

## Results

### Experimental observations of a topological transition in a driven liquid crystal cell

Liquid crystals are composed of rod-like organic molecules^5,13,15^ which, as a result of intermolecular interaction, for specific temperature ranges are arranged to have a similar molecular orientation. This results in a strong anisotropy of all their physical properties, especially optical characteristics^28^. The configuration of lowest energy is reached when all rod-like molecules are aligned along one averaged direction, orientational order without a positional one, denoted by the director vector $\mathbf{n}$^5,13,28^. This state is usually called the nematic phase. In the case of a thin film with negative dielectric anisotropy and molecular anchoring perpendicular to walls of the sample, application of an electric field in the vertical direction leads to the appearance of vortices, umbilic defects or disclination lines^5,13,15^. These topological defects are the result of the competition between elastic and electrical forces. More precisely, the homeotropic anchoring imposes through elastic coupling that the molecules align in the orthogonal (vertical direction) to the walls of the liquid crystal cell. On the other hand, when applying a vertical electric field, the molecules tend to be oriented orthogonally to the electric field (horizontal direction) because the liquid crystal has a negative dielectric constant. For voltages large enough to exceed the elastic resistance, Frédericksz voltage^29^, domains of molecules with different orientations are generated. These different molecular orientation domains are connected by means of vortices. Figure 1a schematically depicts different molecular orientations and how they connect with a vortex. Because the refractive index depends on the molecular orientation^28^ the polarization of the light as it passes through the liquid crystal sample is affected. Considering of the liquid crystal cell between two crossed polarizers allows the detection of the vortices since these correspond to the intercection of two black lines. Figure 1a shows two vortices.

**Figure 2:**
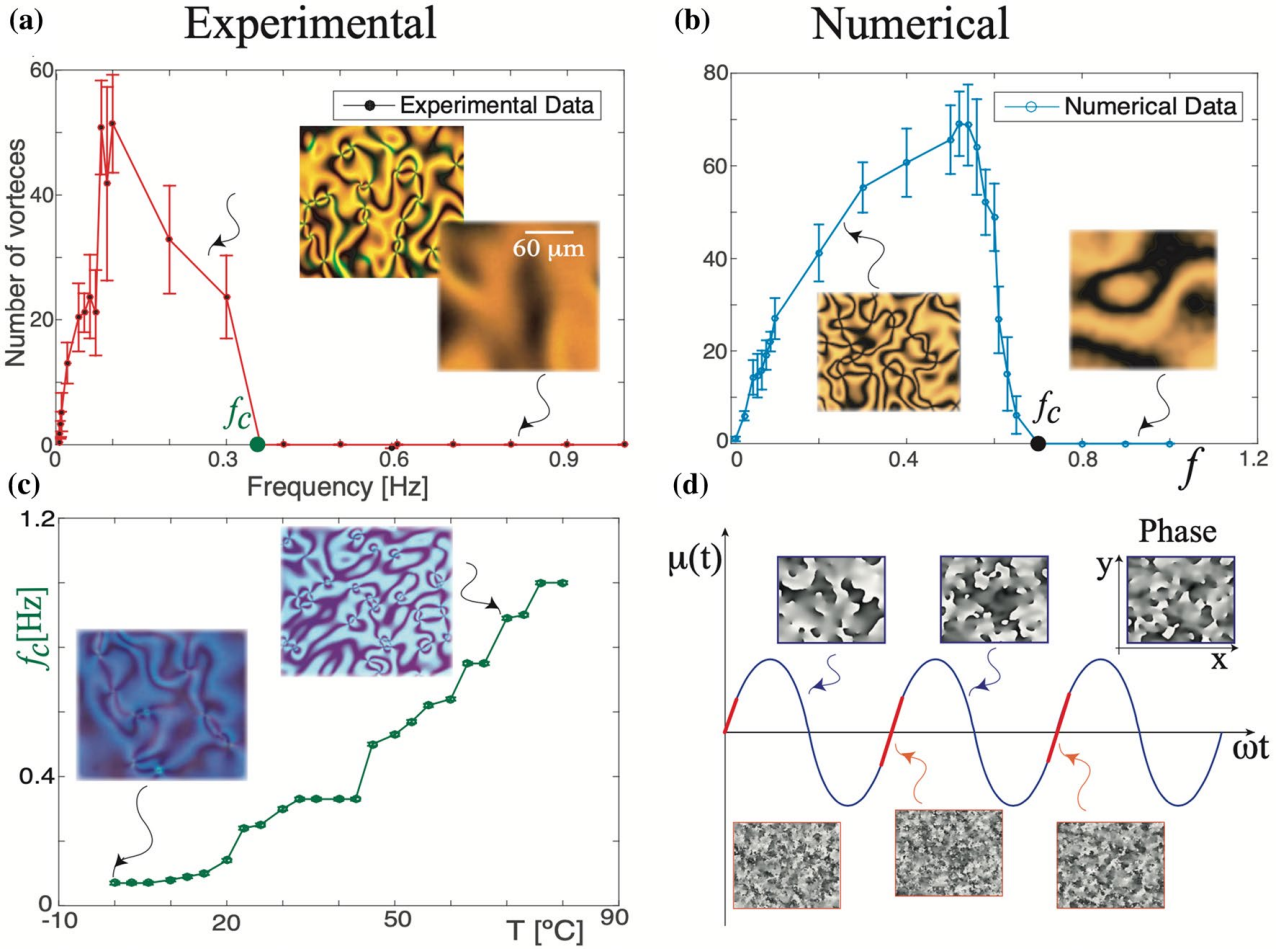
Bifurcation diagram of topological transition out of equilibrium (**a**) experimental and (**b**) numerical using model Eq. (1). The experimental bifurcation diagram is obtained with a sawtooth forcing with a fixed amplitude voltage 15 Vpp. The insets (a) correspond to snapshots obtained in the respective voltage frequency using the crossed polarisers. The insets (**b**) correspond to the polarisation field $\psi =Re(A)Im(A)$ obtained numerically from Eq. (1) at the respective frequency. (**c**) Critical frequency $f_c(T)$ as a function of temperature with a fixed amplitude voltage 15 Vpp. The insets account for the respective snapshots in the different temperatures. (**d**) Evolution of the temporal bifurcation parameter $\mu (t)$ and characterisation of the regimes of creation (red curve) and interaction (blue curve) of vortices. Insets show the amplitude phase $\arctan [Im(A)/Re(A)]$ obtained numerically in the different creation and interaction regimes. From experimental snapshots, both figures were created using Inkscape 1.0.

Figure 1b shows the spatiotemporal evolution of vortex arrangements experimentally observed by applying a voltage $V(t)=V\sin (2\pi f t)$ with a given frequency *f*, i.e, harmonic voltage signal. To avoid charges accumulation effects in the thin film (capacity effects), a high frequency oscillatory electric field (kHz) is usually used. Under these conditions in a homogeneous liquid crystal cell the emergence of gas of disordered vortices is followed by the subsequent annihilation by pairs, and terminates in a homogenous, non-vortex state^10,11,13^. Thus the vortices are a transient phenomenon. Surprisingly, when the frequency of the electric field that we applied to the homogeneous liquid crystal cell decreases to fractions of Hz starting from a critical value of the frequency, the system exhibits a topological transition after which the annihilation and creation are balanced, and the vortices persist (see video 1 in supplementary materials). Hence, the bifurcation parameter of this transition is the frequency *f* of the driving voltage. Figure 2 shows the average number of vortices as a function of frequency counted stroboscopically in each oscillation cycle with the standard deviation determined along the way. This transition is obtained by considering a sawtooth signal for the voltage applied to the sample. From this chart, one deduces that the transition is of continuous nature (supercritical bifurcation) and that there is a critical frequency $f_c$ from which the number of vortices in average becomes permanent over time (frequency $f_c$). We note that as the frequency decreases the number of vortices increases to a particular critical value and subsequently decreases monotonically until it vanishes at low frequencies, which is a manifestation of a sort of resonance for the process of creation and destruction of topological defects. Notice that periodically driven voltage only induces umbilical defects, no other defects are observed. The application of a low-frequency electric field induces charge movements due to the weak anisotropic conductivity of the liquid crystal^5^. The accumulation of charges can induce a molecular reorientation, Carr-Helfrich mechanism^5^, which in turn modifies the interaction between umbilical defects and can even generate a lattice arrangement of them^25^.

Using a thermal control microscope stage, the temperature of the liquid crystal sample can be changed and controlled adequately. When the temperature at which the experiments are made is varied, we observe that critical frequency transition $f_c$ grows monotonically with it as illustrated in Fig. 2c. The tendency to increase the transition frequency at higher temperatures is due to the increasing the rate of vortex creation (fluctuations), while the process of vortices annihilation remains unchanged (deterministic). Therefore, the topological transition induced by temporal voltage modulation is observed throughout the mesophase stability range of the nematic liquid crystal under study.

### Theoretical description of the topological transition

To understand the origin of this topological transition out of equilibrium, we consider a prototype model, the Ginzburg-Landau equation^30^, that describes the emergence of topological defects in fluids, superfluids, superconductors, liquid crystals, chiral magnets, fluidised anisotropic granular matter, and magnetic media^9,30^. The real Ginzburg-Landau equation describe the pattern formation in anisotropic media^31^. Likewise, this model describes vortex solutions in nematic liquid crystal layers with external electric or magnetic forcing and homeotropic boundary conditions^32–35^, and the formation of spiral waves in a nematic liquid crystal subjected to a rotating magnetic^32,34^ or electric field^33^. Note that this Ginzburg-Landau equation with real coefficients is derived from the elastic theory of liquid crystals^32–36^. The order parameter accounts for the balance between the elastic and electric force. Besides, this model describes the process of interaction and annihilation of vortices at constant electric field and temperature^11^. To account for the additional ingredients of the observed topological transition (cf. Fig. 2), we must incorporate the oscillatory nature of the electrical voltage applied to the liquid crystal sample and include the inherent fluctuations due to temperature. This leads to the stochastic Ginzburg-Landau equation with oscillatory coefficients, that is,

1 $$\begin{aligned} \partial _t A=\left[ \mu _0+\gamma \cos (2 \pi f t)\right] A-|A|^2A+\nabla^{2} A+\sqrt{T}\zeta (\vec {r},t) , \end{aligned}$$

where $A(\vec {r},t)$ is a complex order parameter, *t* and $\vec {r}$ describe time and the transversal coordinate vector that characterises the thin film, $\mu _0$ is the uniform bifurcation parameter, $\gamma$ and *f* are the amplitude and the frequency of the forcing, respectively, which account for the oscillatory electric field. The function $\mu (t) =\mu _0+\gamma \cos ( 2 \pi f t)$ is the temporal modulated bifurcation parameter. By $\nabla^{2}$ we denote the Laplace operator. The constant *T* accounts for the thermal intensity and $\zeta (\vec {r},t)$ is a spatiotemporal white noise of zero mean value, $\langle \zeta (\vec {r},t) \rangle =0$, and no spatial or temporal memory. Namely, the stochastic term has the spatiotemporal correlation $\langle \zeta (\vec {r},t) \zeta (\vec {r}',t') \rangle =\delta (\vec {r}-\vec {r}')\delta (t-t')$ where $\delta$ are Dirac delta functions. The real and imaginary part of Α account for the molecular reorientation averaged over the thickness in the *x*- and *y*-direction^32–36^. An umbilic defect corresponds to a zero of the amplitude. In order to compare with the experimental observations obtained by polarised optical microscope, one can consider the polarisation field defined by $\psi (\vec {r},t)=Re(A)Im(A)$^36^, which vanishes when the complex field *A* is purely real or imaginary. Therefore the position of umbilic defects correspond to the intersection of nullclines of $\psi$.

**Figure 3:**
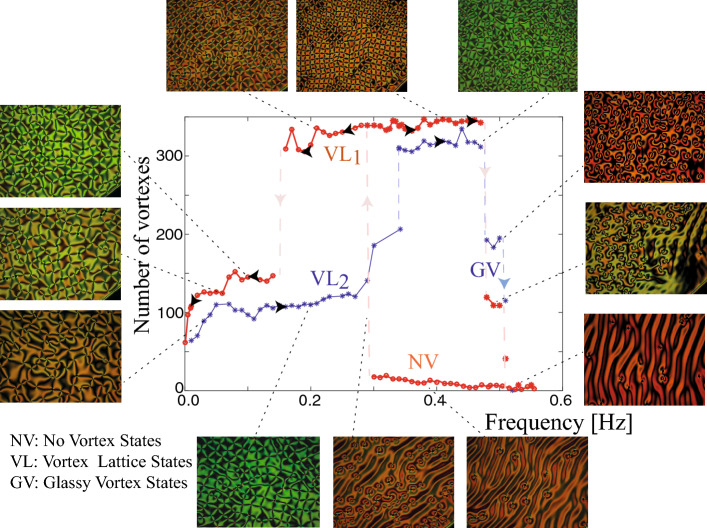
Experimental bifurcation diagram of topological transition out of equilibrium under harmonic forcing. The liquid crystal cell exhibits three states: non-vortex (NV), vortex lattice (VL), and glassy vortex (GV) states. The arrows indicate the direction of increase or decrease of the voltage. The insets show snapshots in the respective parameter ranges. From experimental snapshots, figures were created using Inkscape 1.0.

In the high-frequency regime, $f \rightarrow \infty$, this model becomes the Ginzburg-Landau equation with real coefficients. This equation is characterised by a constant effective bifurcation parameter $\mu _0+3\gamma ^2/2(2\pi f)^2$ obtained through the rapid oscillation method^37^. In this limit the vortices do not persist and the annihilation of the defects of opposite charges dominates their creation^9,11^, since the system tries to optimise the effective free energy. Figure 2b shows this happening for frequency values up to order one. In this regimen, for large enough temporary evolution, the number of vortices on average is zero. By decreasing the frequency further to a critical value $f_c$, the average number of vortices stabilises over time. The topological transition obtained numerically using Eq. (1) has a qualitative behaviour similar to that observed experimentally, see top panels in Fig. 2. Notice that as the frequency decreases ($f<f_c$) the number of vortices increases to a particular critical value and subsequently decreases monotonically until it vanishes at low frequencies, which manifests an excellent qualitative agreement with the experimental observations. Hence, experimentally and numerically a sort of resonance is observed for the process of creation and destruction of topological defects.

The simulation allows us to identify the location of the vortices through $\pm 2\pi$ jumps of the phase of the amplitude. Comparing the evolution of the system and the profile of the bifurcation parameter function $\mu (t)$ two characteristic regions are identified. Namely, a creation and annihilation region. Creation of vortices occurs in the intervals of time where $\mu (t)$ is small and growing (red curve in Fig. 2d), these vortices later interact even when $\mu (t)<0$ (blue curve in Fig. 2d). The region of creation and annihilation are govern by stochastic fluctuations and deterministic evolution, respectively. The vortex creation time interval decreases as the forcing frequency increases and for high frequencies the creation process is inefficient. Hence, the persistence of vortices is a consequence of the balance between the processes of creation (stochastic) and their interaction (deterministic).

### Topological transition with harmonic driven forcing

**Figure 4:**
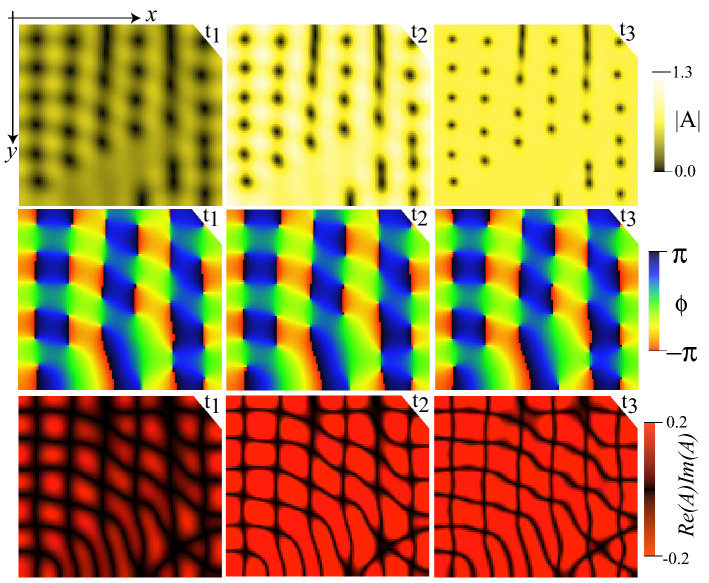
Numerical square vortex lattice. Temporal sequence ($t_1<t_2 <t_3$) of the amplitude module |*A*|, phase $\phi =arctan[Im(A)/Re(A)]$, and polarisation field *Re*(*A*)*Im*(*A*) of model Eq. (2) with inertia and anisotropic coupling over a period, by $\mu _0=0.6$, $T=0.03$, $\lambda =1.4$, $\delta =0.3$, $\gamma =3$, and $f=0.1$. From numerical simulations, figure was created using Inkscape 1.0.

In experiments we have implemented various types of periodic forcing among them harmonic, sawtooth, or square profiles and we have found, somewhat unexpectedly, different types of responses resulting in diverse transitions. As we have mentioned, low-frequency voltages can induce charge movements that, in turn, induce molecular reorientation, Carr-Helfrich mechanism^5^. Hence, different types of driven voltages can induce different charge motions. In the case of a square profile signal, we have observed a continuous or supercritical topological transition (see Fig. 2a). Changing to a harmonic signal, we have detected a discontinuous transition with the non-vortex state being replaced by a vortex lattice with a square crystalline structure. Figure 3 shows a square vortex lattice and its respective bifurcation diagram corresponding to the out of equilibrium counterpart of the Abrikosov lattice^38,39^. The vortex lattice is not hexagonal like the one of Abrikosov as a consequence of the asymmetry between the opposite charges^35^. The model Eq. (1) only accounts for the topological transition from disordered vortices to non-vortex state. The origin of these square vortex lattices is probably associated with the coupling of elastic deformations, charge, and fluid motions. To account qualitatively of this coupling, we include phenomenologically in the model Eq. (1) inertia and anisotropic effects, that is, a second temporal derivative of amplitude A. Simulations of this model show the emergence of a square lattice, as seen in Fig. 4. The Ginzburg-Landau Eq. (1) is a model valid close to the reorientational transition^34,36^. Its derivation is based on the assumption of slowly varying amplitude; however, when the system is periodically forced, the first and second temporal variations can be of the same order. Hence, the inertia term phenomenologically accounts for the effects of movements of charges and liquid crystal inside the cell.

When decreasing the frequency, the square lattice undergoes a subcritical bifurcation leading to a square lattice of higher wavelength (see Fig. 3 and supplementary video 2). Increasing the frequency further the square lattice transitions to a glassy state (cf. Fig. 3 and supplementary video 3), in which the vortex structure does not have a precise unit cell. For even higher frequencies the system returns to the non-vortex state. Figure 3 summarises the complexity of the topological transitions in the liquid crystal cell maintained out of equilibrium at room temperature. We speculate that the origin of the periodic structures we have discovered may be associated with the interaction between the vortices or the excitation of stationary waves^51^. However, experimentally we did not detect waves. A precise understanding of this is an open problem.

## Discussion

Topological defects in liquid crystals are natural elements used for the generation of optical vortices^18,22,40–42^. As a matter of fact, optical vortices have attracted attention for their diverse photonic applications ranging from optical tweezers^43,44^, quantum computation^45^, enhancement of astronomical images^46^. In all these applications, optical vortex lattices are always involved and necessary^47–50^. These vortex lattices require sophisticated and complex experimental setup. Instead, vortex lattices that we observe emerge spontaneously in simple liquid crystal cells under the influence of an oscillatory voltage that do not require a complex structure of electrodes, inhomogeneities, applications of thermal gradients, combined forcing of electric and magnetic fields, or photosensitive walls.

In conclusion, we have shown that exotic states of matter, topological lattices, with injection and dissipation of energy by means of oscillatory forcing. In a nematic liquid crystal cell under the influence of a low frequency oscillatory electric field, we have observed transitions from a non-vortex state to a state in which vortices persist. The bifurcation diagrams and critical frequency as a function of temperature is revealed. Depending on the frequency and type of the forcing (harmonic, sawtooth, or square profiles), the vortices self-organise, forming square lattices, glassy states, and disordered vortex structures. Continuous topological transitions to disordered vortex structures are observed for sawtooth-type forcing (cf. Fig. 2). These transitions can be understood as the balance between stochastic vortex creation (thermal fluctuations) and deterministic interaction (vortex annihilation). Unexpectedly, when considering harmonic forcing this scenario change drastically, the topological transitions are characterised by the emergence of regular vortex lattices (discontinuous transitions), like square lattices (see Fig.3). The emergence of these vortex lattices may be a result of the charge movements due to the weak anisotropic conductivity of the liquid crystal (Carr-Helfrich mechanism)^5^ and couplings with standing waves^51^. However, we observe complex structures of vortex lattices like glassy and disorder states (see Figs. 2 and 3), which cannot be described as couplings of standing waves nor self-organisation of charges. The physical mechanism of the complex vortex structures observed is unknown. Because the phenomenon reported here is qualitatively well described by a universal model Eq. (1) and its respective extension Eq. (2), we expect that any temporally modulated vectorial field system of low dimensionality can exhibit topological transitions out of equilibrium. Note that the amplitude equations considered are perhaps the minimal models that describe topological transitions and the emergence of vortex lattices. The inertial amplitude equation, model (2), is a phenomenological description. The study of re-orientational instability considering first-principle models that include the director and the fluid dynamics, nematodynamics and hydrodynamics, is a strategy to derive inertial amplitude equation, work in this direction is in progress. The characterisation of the critical frequency and voltage as a function of liquid crystal features and cell configuration is an open question. Work in this direction also is in progress. Furthermore, these findings could be a starting point for understanding and controlling the exotic states of matter out of equilibrium by means of the temporal modulation of parameters. Because vortex lattices emerge spontaneously in single cells subjected to alternative low-frequency voltages, it opens up the possibility of new and fresh applications of the generation of optical vortices.

## Methods

### Experimental description of the setup

Figure 1a shows a schematic representation of the experimental setup. It consists of a liquid crystal cell composed of two glass slabs with 600 mm$^2$ of cross-section separated by a distance of 15 $\upmu$m, a thin film of a transparent conductor, indium tin oxide (ITO), and a thin film of transparent polyimide that has been deposited on each of the interior walls. Transparent conductors are used as electrodes. By rotation and evaporation, the polyimide molecules are oriented orthogonal to the surface, this layer allows the liquid crystal molecules anchoring orthogonal to the surfaces^52^, homeotropic anchoring. This cell 5B100A150UT180 manufactured by Instec, contains glass beads as spacers. It is filled by capillarity with BYVA-01 (Instec) nematic liquid crystal that has negative anisotropy, $\epsilon _a=-4.89$ at room temperature. An external electric field is applied in the vertical direction (z-axis) using a sinusoidal, sawtooth, or square voltage with amplitude 15 Vpp with low frequency. This voltage is produced by a function generator (Agilent 33521A) with a high voltage amplifier (Tabor Electronics 9200). The imaging system used is an Olympus BX51 microscope equipped with linear cross polarisers. The light from the microscope condenser illuminates the cell mounted on the microscope stage, and a CMOS camera (Thorlabs DCC1645C) is used to capture images. For studying thermal effects we used Leica DM2700 P microscope equipped with LTS420 hot stage.

### Numerical simulations

Numerical simulations of model Eq. (1) were implemented using a finite differences code with Runge–Kutta order-4 algorithm, with a 200$\times$200 points grid, spacing $dx=0.5$, and temporal increment $dt=0.02$. Numerical simulations are performed with periodic boundary conditions and with an initial condition $A = 0$. The stochastic noise $\zeta (\vec {r},t)$ is generated through the Box-Muller transform of a uniform random number generator. Equation (1) with inertia and anisotropic effects reads

2 $$\begin{aligned} \partial _{tt} A+\lambda \partial _t A=\left[ \mu _0+\gamma \cos (2 \pi f t)\right] A-|A|^2A+ \nabla^{2} A +\delta \partial _{\eta ,\eta }\bar{A}+\sqrt{T}\zeta (\vec {r},t), \end{aligned}$$

where $\lambda$ accounts for the rotational viscosity, $\delta$ stands for the difference of elastic constants^32–35^, the operator $\partial _{\eta ,\eta }=\partial _{xx}-\partial _{yy}+2i\partial _{xy}$ describes the asymmetric coupling, and $\bar{A}$ is the complex conjugate of *A*. The results presented in Fig. 4 consider the same algorithm, boundary and initial conditions used in Eq. (1).

## Supplementary information

Supplementary material 1

Supplementary material 2

Supplementary material 3

Supplementary material 4
