# Supplementary material for: Topological transitions in an oscillatory driven liquid crystal cell
Source: Sci Rep. 2020 Nov 9;10:19324. doi: 10.1038/s41598-020-75165-8 (PMC7653948; doi:10.1038/s41598-020-75165-8)
Supplement: Supplementary file 1 — Supplementary material 1 [file 41598_2020_75165_MOESM1_ESM.docx]

**Supplementary Information to**

**Exotic states of matter in an oscillatory driven liquid crystal cell**

Marcel G. Clerc,^1^ Michal Kowalczyk,^2^ and Valeska Zambra^1^

^1^Departamento de Física and Millennium Institute for Research in Optics,

FCFM, Universidad de Chile, Casilla 487-3, Santiago, Chile.

^2^Departamento de Ingeniería Matemática and Centro de Modelamiento Matemático (UMI 2807 CNRS), Universidad de Chile, Casilla 170 Correo 3, Santiago, Chile.

**Video 1:** Vortices evolution close to the topological transition at room temperature (T=24 ºC) with a sawtooth voltage of an amplitude 15 Vpp and frequency 0.3 Hz. The observed region corresponds to 1.394 mm^2^. In each oscillation cycle, the vortices appear in different regions and tend to annihilate with vortices of opposite charges. Hence, the system exhibits, on average, a nonnull number of vortices per vortex cycle.

**Video 2:** Vortex lattice observed in a nematic liquid crystal cell at room temperature (T=24 ºC) with a harmonic voltage of an amplitude 15 Vpp and frequency 0.2 Hz. The monitoring region corresponds to 1.394 mm^2^. In each oscillation cycle, a stationary square vortex lattice is observed, affected by the presence of glass beads.

**Video 3:** Glassy vortex lattice observed in a nematic liquid crystal cell at room temperature (T=24 ºC) with a harmonic voltage of an amplitude 15 Vpp and frequency 0.5 Hz. The monitoring region corresponds to 1.394 mm2. A disordered vortex lattice is observed, which vortex positions alternate every two cycles (parametric vortex lattice).
